# Prostate MRI quality improvement: a Roadmap from the ESUR Prostate MRI Working Group

**DOI:** 10.1007/s00330-026-12395-w

**Published:** 2026-03-13

**Authors:** Adriano B. Dias, Jelle Barentsz, Clare Allen, Ronaldo Hueb Baroni, Hanna Falińska, Caterina Gaudiano, Rossano Girometti, Rajan T. Gupta, Fredrik Jäderling, Daniel Junker, Guido Matthias Kukuk, Ana Sofia L. Moreira, Vibeke Løgager, Philippe Puech, Andrei S. Purysko, Johannes Uhlig, Stephan Ursprung, Geert Villeirs, Jonathan Richenberg, Francesco Giganti

**Affiliations:** 1 University Medical Imaging Toronto, Joint Department of Medical Imaging (University Health Network/Sinai Health System/Women’s College Hospital) and Department of Medical Imaging, University of Toronto, Toronto, Canada; 2 Department of Medical Imaging, Andros Clinics, Arnhem, The Netherlands; 3 Department of Radiology, University College London Hospitals NHS Foundation Trust (UCLH), London, UK; 4 Department of Radiology, Hospital Israelita Albert Einstein, São Paulo, Brazil; 5 Department of Radiology, Affidea Poland, Warsaw, Poland; 6 Department of Nuclear Medicine, Military Institute of Medicine–National Research Institute, Warsaw, Poland; 7 Department of Radiology, IRCCS Azienda Ospedaliero-Universitaria di Bologna, Bologna, Italy; 8 Institute of Radiology, Department of Medicine (DMED), University of Udine, Udine, Italy; 9 University Hospital Santa Maria della Misericordia, Azienda Sanitaria Universitaria Friuli Centrale (ASUFC), Udine, Italy; 10 Department of Radiology, Duke University School of Medicine, Durham, NC USA; 11 Duke Cancer Institute, Center for Prostate & Urologic Cancers, Durham, NC USA; 12 Department of Radiology, Capio S:t Göran’s Hospital, Stockholm, Sweden; 13 Department of Molecular Medicine and Surgery, Karolinska Institutet, Stockholm, Sweden; 14 Department of Radiology, Community Hospital Hall in Tirol (Tirol Kliniken), Hall in Tirol, Austria; 15 Department of Radiology, Medical University of Innsbruck, Innsbruck, Austria; 16 Department of Radiology, Kantonsspital Graubünden, Chur, Switzerland; 17 Department of Radiology, Unidade Local de Saúde do Algarve (ULS Algarve), Faro, Portugal; 18 Department of Radiology, Copenhagen University Hospital–Herlev and Gentofte, Herlev, Denmark; 19 Department of Radiology, CHU Lille (Lille University Hospital), Lille, France; 20 University of Lille, Lille, France; 21 Imaging Institute, Abdominal Imaging Section and Department of Nuclear Medicine, Diagnostics Institute, Cleveland Clinic, Cleveland, OH USA; 22 Departments of Urology and Colorectal Surgery, Cleveland Clinic, Cleveland, OH USA; 23 Department of Clinical and Interventional Radiology, University Medical Center Göttingen (UMG), Göttingen, Germany; 24 Department of Diagnostic and Interventional Radiology, Tübingen University Hospital, Eberhard Karls University Tübingen, Tübingen, Germany; 25 Department of Radiology, Netherlands Cancer Institute (NKI), Amsterdam, The Netherlands; 26 Department of Radiology and Nuclear Medicine, Ghent University Hospital (UZ Gent), Ghent, Belgium; 27 Department of Imaging, University Hospitals Sussex NHS Foundation Trust, Brighton and Sussex Medical School, Brighton, UK; 28 Division of Surgery & Interventional Science, University College London (UCL), London, UK

**Keywords:** Magnetic resonance imaging, Prostatic neoplasms, Quality control

## Abstract

**Abstract:**

Prostate magnetic resonance imaging (MRI) has become a crucial tool in diagnosing and managing prostate cancer, mainly by helping to avoid unnecessary biopsies and enhancing the detection of clinically significant disease. However, its clinical usefulness is often limited by wide variation in how images are acquired, interpreted, and reported worldwide. This inconsistency affects diagnostic accuracy and patient outcomes. In response, the Quality Improvement Subgroup of the European Society of Urogenital Radiology (ESUR) Prostate MRI Working Group has created a practical, three-step quality-improvement framework aimed at standardising and improving prostate MRI practices. This framework consists of: Step 1: ‘Build it right’, establishing a foundation of technical excellence through adherence to the Prostate Imaging Reporting and Data System (PI-RADS) technical standards, objective quality assessment using the Prostate Imaging Quality (PI-QUAL) score, and systematic artefact reduction. Step 2: ‘See it right’, emphasising interpretive excellence via structured training, institutional quality assurance metrics, and multidisciplinary collaboration. Step 3: ‘Improve and innovate’, promoting continual refinement through emerging technologies such as AI-driven assessment, deep learning reconstruction, and remote supervision. By incorporating this structured approach into daily practice, this framework aims to ensure that prostate MRI consistently fulfils its promise of accurate, reproducible, and patient-centred care. A coordinated effort towards international implementation, benchmarking, and outcome-based validation represents the next critical step to maximise global impact.

**Key Points:**

***Question*** *Wide variation in prostate MRI acquisition, image quality, and reporting undermines diagnostic accuracy. A structured roadmap is needed to ensure consistent quality and reproducible practice.*

***Findings*** *The ESUR Prostate MRI Working Group outlines a three-step framework — ‘Build it right’, ‘See it right’, ‘Improve and innovate’ — to standardise acquisition, interpretation, and quality assurance.*

***Clinical relevance*** *Applying this roadmap in clinical practice aims to enhance diagnostic confidence and promote consistent, high-quality prostate cancer care across diverse healthcare settings.*

## Introduction—why prostate MRI quality matters

Prostate MRI is key in the diagnosis and management of prostate cancer (PCa). Its high negative predictive value has reduced unnecessary biopsies and minimised the detection of clinically insignificant prostate cancer [1–3]. As a method for lesion targeting, it improves the detection of clinically significant (cs)PCa compared with systematic biopsy alone [4]. Its benefits as a diagnostic tool for high-risk individuals have prompted consideration of MRI as a potential screening tool for larger populations [5].

With the establishment of MRI in the prostate cancer pathway, the focus must now shift to ensuring quality and driving quality improvement. Consistency in acquiring diagnostic studies and reproducibility in reporting are *sine qua non* for extending MRI across continents and patient populations—whether applied pre-diagnosis, during active surveillance, post-treatment, or in a screening programme [6–10]. High standards in image acquisition, interpretation, reporting, training, certification, accreditation, and protocols are essential to ensure radiologists are well qualified and imaging results can be reliably used in clinical decision-making [11–15]. However, despite established PI-RADS guidelines, practical implementation remains highly variable, highlighting the need for a unified roadmap that addresses real-world integration and continuous quality improvement.

### The 3-point scoring system

The importance of quality in prostate MRI is supported by increasing evidence of its impact on diagnostic confidence [16–23]. The Prostate Imaging Quality (PI-QUAL) [24] framework—recently updated to version 2 [25], which uses a three-point scale to rate overall image quality—provides an objective and reproducible method for assessing image quality, enabling comparison between institutions and readers, with good inter-reader agreement [26, 27]. It applies to both multiparametric (mpMRI) and MRI without intravenous contrast medium, or biparametric (bpMRI).

The large multicentre GLIMPSE study [28], which included 41 centres across 18 countries within the PRIME trial [29], demonstrated substantial variability in adherence to PI-RADS v2.1 technical standards [28]. After minor protocol changes, 97% of scanners achieved optimal diagnostic quality, underscoring the importance of continuous improvement and adherence to established standards.

**Fig. 1:**
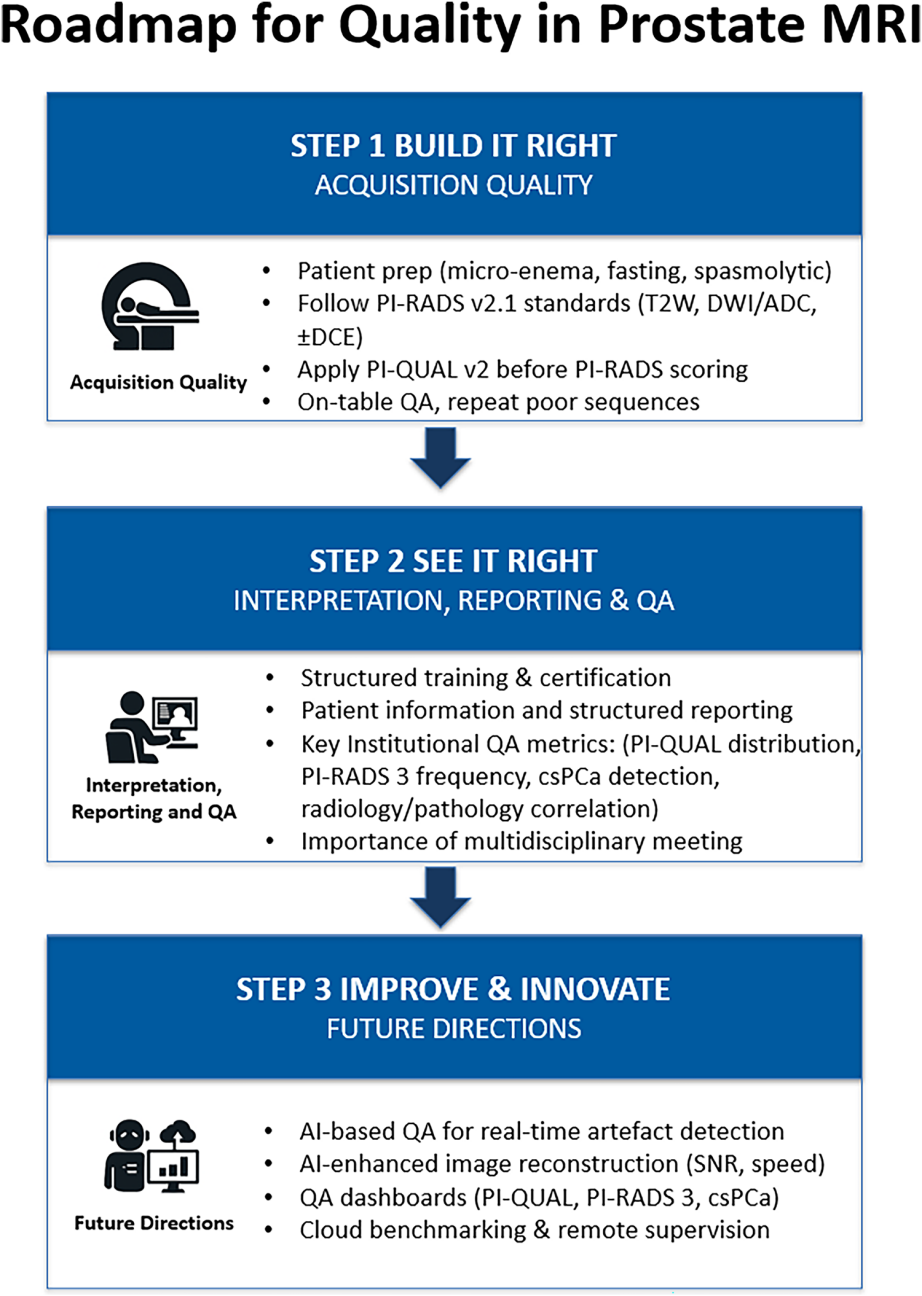
Roadmap for quality in prostate MRI. A three-step framework from the ESUR Prostate MRI Working Group emphasises: (1) Build it right—acquisition quality through patient preparation, adherence to PI-RADS standards, and PI-QUAL scoring; (2) See it right—interpretation and reporting supported by structured training, templates, and institutional QA metrics; and (3) Improve and innovate—future directions with AI-based QA, image reconstruction, and cloud-based benchmarking. PI-RADS, Prostate Imaging-Reporting and Data System; PI-QUAL, Prostate Imaging Quality; DWI, diffusion-weighted imaging; ADC, apparent diffusion coefficient; DCE, dynamic contrast-enhanced imaging; csPCa, clinically significant prostate cancer; QA, quality assurance; SNR, signal-to-noise ratio

Given these premises, this Special Report, prepared by the Quality Improvement Subgroup of the European Society of Urogenital Radiology (ESUR) Prostate MRI Working Group, provides a step-by-step framework (Step 1: ‘Build it right’; Step 2: ‘See it right’ and Step 3: ‘Improve and innovate’) (Fig. 1) addressing acquisition, interpretation, automation, and control to support and maintain excellence in prostate MRI. This work represents a narrative review developed through expert consensus and synthesis of current evidence and guidelines.

## Step 1: Build it right—acquisition quality

### Foundations: PI-RADS v2.1 minimum technical standards

The PI-RADS v2.1 document outlines the minimum technical requirements for prostate MRI to ensure reproducibility and accuracy. These include high-resolution T2-weighted imaging in at least two planes (axial + coronal and/or sagittal), axial diffusion-weighted imaging (DWI) with high b-value (≥ 1400 s/mm²), an apparent diffusion coefficient (ADC) map, and axial dynamic contrast-enhanced imaging for multiparametric MRI (mpMRI). These technical standards represent a broad international consensus, jointly developed and endorsed by major radiological societies including ESUR and the American College of Radiology (ACR), and form the basis for clinical guidelines and accreditation programmes in multiple continents. 1.5-T scanners can be used, although 3 T is preferred due to its higher signal-to-noise ratio (SNR). However, gradient strength is equally important, as a high gradient strength is required for good DWI quality. The PI-RADS v2.1 minimum recommended acquisition parameters are summarised in Table 1.

**Table 1:** Prostate MRI acquisition protocol (aligned to PI-RADS v2.1)

Priority	Sequence	Coverage and plane	Technical specifications	Notes
Required (mpMRI and bpMRI)	Axial T2W (2D FSE/TSE)	Prostate and seminal vesicles; straight axial or oblique axial perpendicular to gland long axis^¥^	Slice 3 mm*, no gap; FOV 12–20 cm; in-plane ≤ 0.7 mm (phase) × ≤ 0.4 mm (frequency)	Core sequence; at least one orthogonal plane also required
Recommended	Coronal T2W	Orthogonal to axial	Slice 3 mm	Improves anatomic assessment
Recommended	Sagittal T2W	Orthogonal to axial	Slice 3 mm	Complements axial/coronal planes
Required (mpMRI and bpMRI)	DWI + ADC (spin-echo EPI, spectral fat sat)	Same axial plane as T2W	TE ≤ 90 ms; TR ≥ 3000 ms; slice ≤ 4 mm*, no gap; FOV 16–22 cm; in-plane ≤ 2.5 mm; ADC calc b-values: low 0–100 s/mm² (prefer 50–100) + intermediate 800–1000; high b-value ≥ 1400 s/mm² (acquired or calculated)	DWI/ADC must match T2W plane; high b-value mandatory
Required (mpMRI only)	DCE (3D T1W GRE preferred)	Same plane as T2W/DWI; prostate and SV	Slice 3 mm, no gap; in-plane ≤ 2 mm; temporal resolution ≤ 15 s; observation ≥ 2 min; GBCA dose 0.1 mmol/kg; injection rate 2–3 mL/s	DCE useful for upgrading PZ PI-RADS 3
Optional	Large-FOV sequence	Pelvis to aortic bifurcation	Large-FOV T1W (pre-/post-contrast per practice) or 3D T2W with isotropic voxels	Ensures LN assessment to aortic bifurcation

Minimum required, recommended, and optional sequences for prostate MRI are summarised. Parameters apply to both biparametric MRI (bpMRI) and multiparametric MRI (mpMRI), with DCE sequences included only in mpMRI. Adherence to these standards is essential to ensure diagnostic adequacy and avoid non-diagnostic examinations (i.e., PI-QUAL score of 1). These parameters are based on broad international consensus recommendations from the PI-RADS Steering Committee, which includes representatives from the ESUR, ACR, and the AdMeTech Foundation

*T2W* T2-weighted, *DWI* diffusion-weighted imaging, *ADC* apparent diffusion coefficient, *DCE* dynamic contrast-enhanced, *bpMRI* biparametric MRI, *mpMRI* multiparametric MRI, *ST* slice thickness, *FOV* field of view, *GRE* gradient echo, *PI-QUAL* prostate imaging quality score, *PI-RADS* Prostate Imaging Reporting and Data System, *LN* lymph nodes *EPI* Echo Planar Imaging, *TR* Repetition time, *TE* Echo Time *FSE* Fast Spin Echo *TSE* Turbo Spin Echo

**ESUR Working Group comments:**

¥ Once a T2W plane is chosen, the same orientation should be used for all sequences (DWI, DCE) to maintain consistency

* For T2W and DWI, slice thickness must be exactly 3.0 mm. Thinner 2D slices (e.g., 2.9 mm) do not meet PI-RADS standards. For T2W, optional 3D isotropic acquisitions may be reconstructed into 3 mm planes, but these should complement rather than replace mandatory 2D FSE/TSE sequences. Consistent slice thickness across all sequences (T2W, DWI and DCE) is critical to avoid missed small lesions and minimise misregistration

### Going beyond the minimum: PI-QUAL for objective quality scoring

**Fig. 2:**
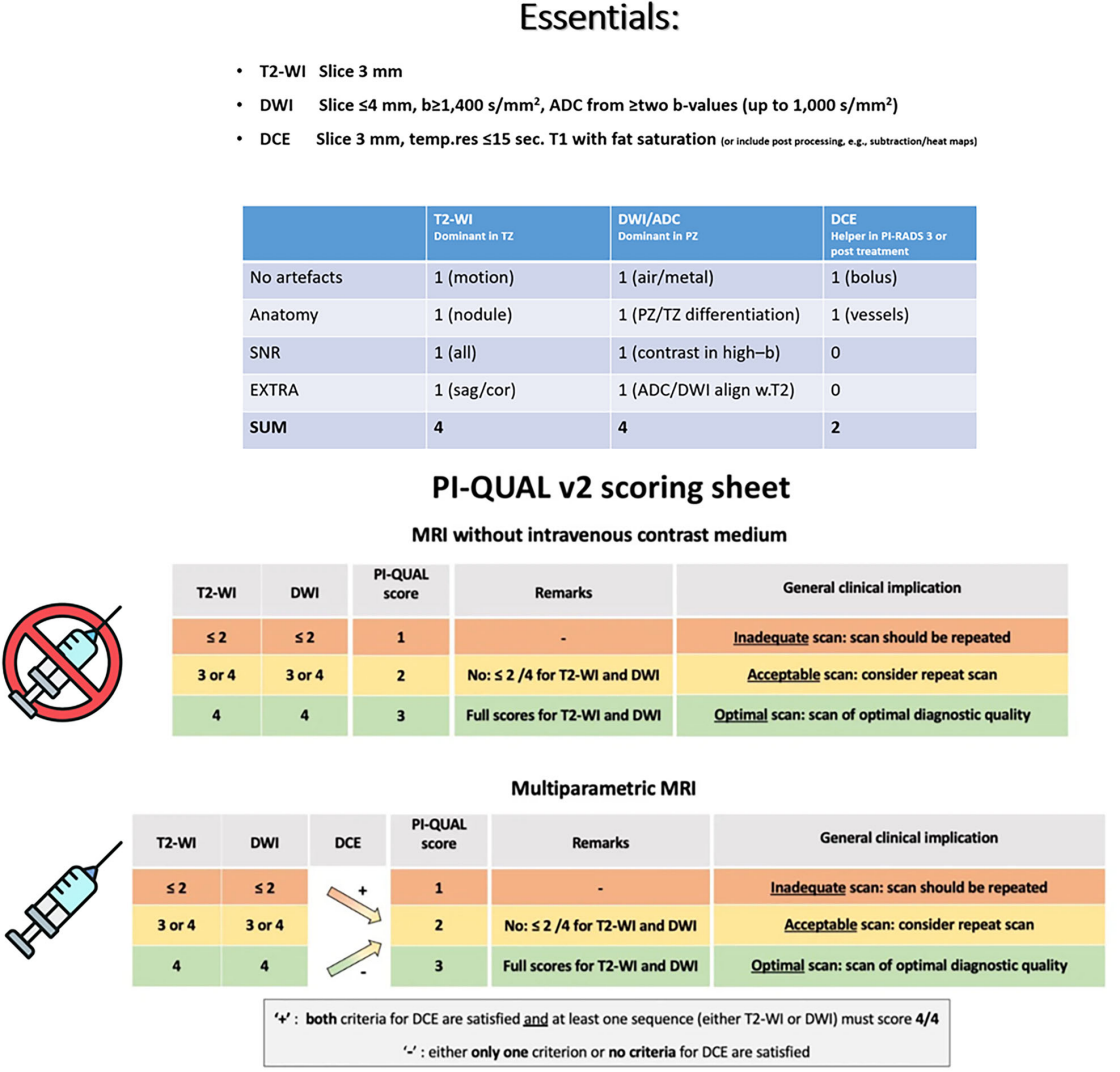
Adapted schematic representation of the PI-QUAL v2 scoring system for prostate MRI quality assessment (adapted from de Rooij et al [25]). The top section summarises the Essential acquisition criteria, which define the minimum technical standards for T2-weighted imaging (T2-WI), diffusion-weighted imaging/apparent diffusion coefficient (DWI/ADC), and dynamic contrast-enhanced (DCE) sequences. If these essentials are not met, the sequence automatically scores 0 points for T2-WI and DWI/ADC, or “–” for DCE. In this schematic, a subtraction approach is applied, where missing or inadequate features are deducted to yield the cumulative sequence score. The bottom panels illustrate interpretation using the PI-QUAL v2 colour-coded scoring sheet. For MRI without intravenous contrast (top table), the overall PI-QUAL score is derived from T2-WI and DWI only. For multiparametric MRI (mpMRI, bottom table), the DCE sequence contributes if both DCE criteria are fulfilled; otherwise, it is scored as “–” and does not upgrade the exam. Scans are then classified as inadequate (red: repeat required), acceptable (orange: usable but repeat may be considered), or optimal (green: diagnostic quality). This framework provides a practical way to apply PI-QUAL in daily practice: assess essentials, subtract missing features, and use the colour-coded scoring sheet for a clear, clinically actionable quality classification. T2-WI, T2-weighted imaging; DWI, diffusion-weighted imaging; ADC, apparent diffusion coefficient; DCE, dynamic contrast-enhanced; mpMRI, multiparametric MRI

The PI-QUAL v2 score [25] (up to 3 points) should be systematically applied in clinical practice (Fig. 2). The score for any examination combines technical and visual criteria to grade the visibility of normal structures rather than tumour. Only PI-QUAL scores of 2 or 3 should be considered diagnostic. A lower score (i.e., score 1) indicates insufficient diagnostic quality, and a PI-RADS (or Likert) score should not be assigned.

It is important to recognise that the PI-QUAL score should inform rather than dictate clinical decision-making. For example, an examination may score PI-QUAL 1, yet a large lesion remains visible and could still be targeted. In such cases, a pragmatic approach is to report both the PI-QUAL score and a brief subjective statement on adequacy. Repeat imaging is generally advised unless the limitation is irrecoverable (e.g., bilateral hip prostheses), in which case the report should clearly document the cause and any mitigation strategies (e.g., sequence orientation changes, complementary sequences).

### Multifaceted approach to a high-quality study

High-quality prostate MRI requires a coordinated approach that combines patient preparation, technical choices, team expertise, and workflow optimisation:

- Patient preparation: Measures to reduce rectal gas and suppress bowel motion [30–32].
- Technical choices: A 3-T scanner without an endorectal coil is preferred, provided susceptibility artefacts are mitigated. A well-calibrated 1.5-T scanner can outperform a poorly configured 3-T system, and 1.5 T may benefit patients with metallic hip prostheses or pacemakers [33, 34]. The GLIMPSE study demonstrated that protocol optimisation and quality standards are more important for diagnostic quality than field strength [28].
- Non-contrast MRI is increasingly adopted. This is supported by multiple studies, including the Level I PRIME trial, which showed that, when image quality is high and the reporting radiologists are experienced, bpMRI is non-inferior to mpMRI for detecting csPCa [29], with advantages in scan time, cost, and patient tolerance. The authors and the ACR–PI-RADS Steering Committee highlight, however, that contrast can only be omitted if image quality and interpretive accuracy are unequivocally sufficient.
- Workflow and team expertise: Please see the “Institutional quality assurance frameworks: from benchmarks to multidisciplinary integration” section for a detailed account.
- Balancing the MRI Quality Triangle: Achieving optimal quality involves balancing SNR, spatial resolution, and scan time, as improvements in one parameter affect the others. Compromises should be guided by clinical priorities (Fig. E1).

Key artefacts and their solutions, along with practical recommendations before, during, and after the examination, are summarised in Table 2. These measures help minimise artefacts that can compromise prostate MRI quality. Figures 3, 4 and E2–E8 illustrate practical examples of solutions to improve image quality.

**Fig. 3:**
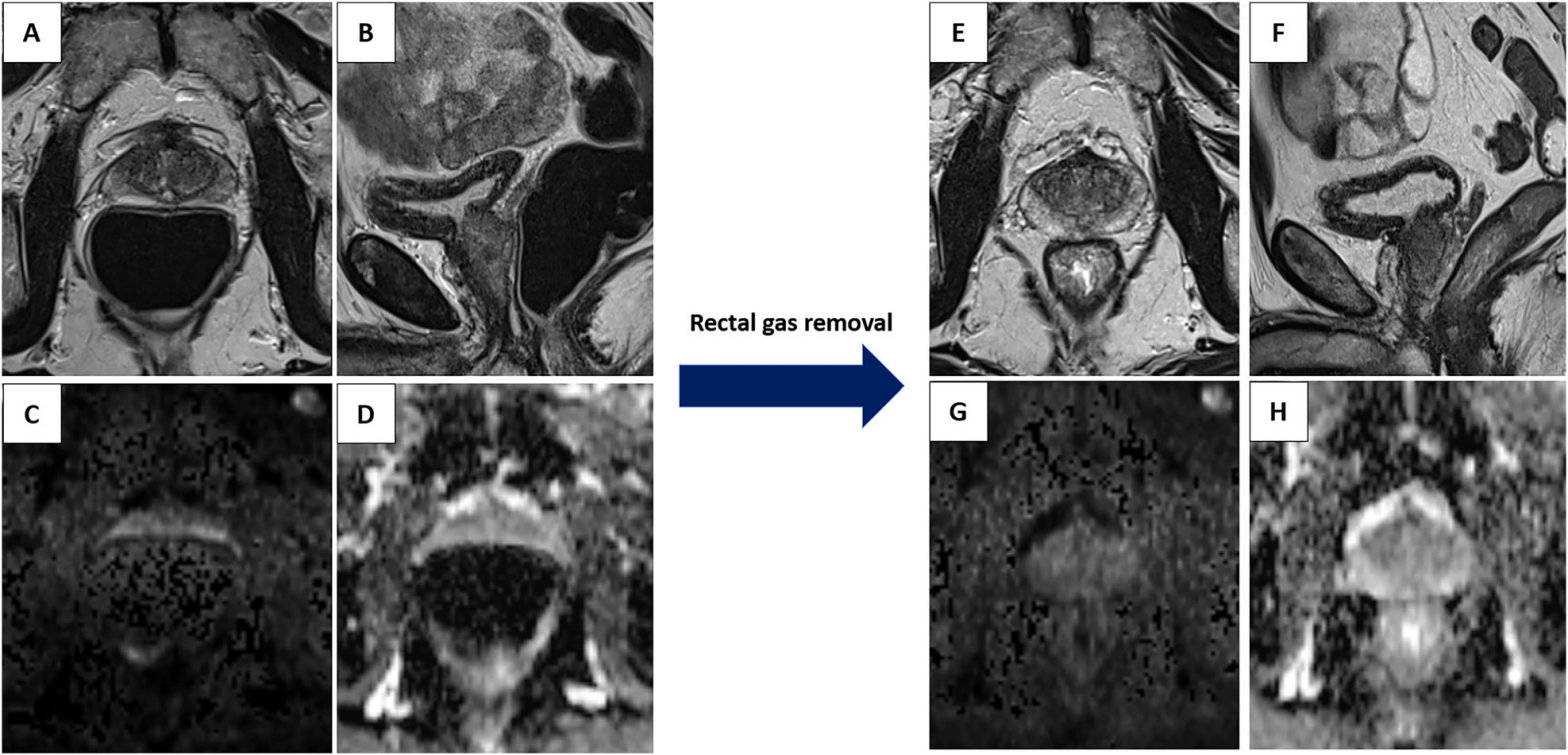
Effect of rectal gas and its removal on prostate MRI quality. **A**–**D** Axial and sagittal T2-weighted images (top) with corresponding DWI and ADC maps (bottom) show marked rectal gas artefact, obscuring the posterior prostate and limiting assessment. **E**–**H** After rectal gas evacuation using a rectal catheter, both T2-weighted and diffusion images demonstrate substantial improvement, enabling confident gland evaluation. This case illustrates how rectal gas can significantly degrade prostate MRI and how its removal—whether by rectal catheter or simple evacuation—can restore diagnostic confidence

**Fig. 4:**
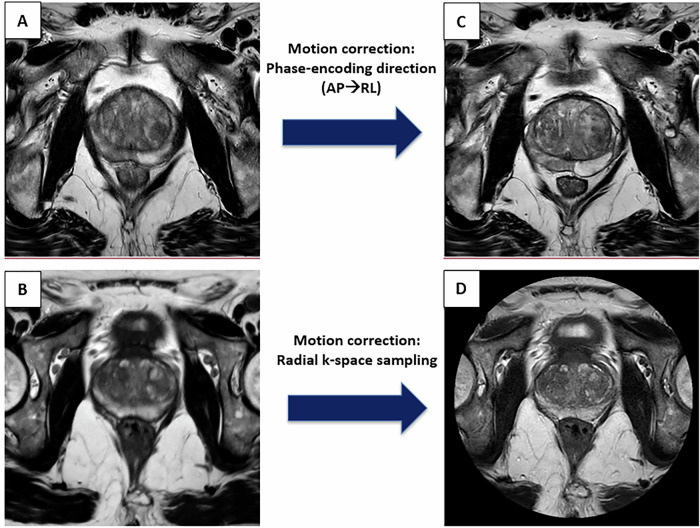
Effect of motion correction techniques on axial T2-weighted imaging. **A**, **B** Motion-related ghosting artefacts are seen as blurring and loss of sharp prostate margins. **C** Switching the phase-encoding direction from anterior–posterior (AP) to right–left (RL) reduces rectal motion artefacts, improving lesion conspicuity. **D** Radial k-space sampling (MultiVane XD) further mitigates ghosting by oversampling the k-space centre and redistributing motion effects, resulting in superior image quality. This example highlights how advanced acquisition strategies can substantially reduce motion-related artefacts in prostate MRI

**Table 2:** Optimising prostate MRI quality: artefacts, patient factors, and workflow strategies

Stage/category	Issue/artefact	Affected sequences	Practical solutions	Rationale
Before scan (patient-related factors)	Rectal gas/stool	DWI (± T2W)	Empty bowel prior to exam (Fig. 3); micro-enema (Fig. E2); acquire DWI early if sagittal T2W acceptable	Reduces susceptibility distortion
	Bowel motion	T2W DWI, DCE	Diet modifications; fasting; avoid caffeine/nicotine; spasmolytics if not contraindicated	Minimises motion artefacts
	Sexual activity (optional)	T2W, DWI	Abstinence for 2–3 days may reduce seminal vesicle fluid variation and improve signal in the PZ (Fig. E3)	Standardises signal in SV/PZ
	Anxiety/claustrophobia	T2W, DWI, DCE	Calm staff interaction; consider sedation if severe	Reduces motion, improves compliance
During scan (technical/workflow)	Aliasing (wrap-around)	Any	Increase FOV; use “no-phase-wrap” or “phase-oversampling” techniques; apply saturation bands outside FOV	Avoids wrap-around artefacts
	Noise	Any	Increase signal averaging; reduce matrix size	Improves SNR
	Chemical shift	Spin-echo, GRE, Dixon	Change frequency/phase direction; increase bandwidth	Reduces misregistration
	Signal distribution (low SI in the prostate region)	Any	Use homogeneity correction solutions (Fig. E4)	Improves uniformity of signal
	Breathing artefacts (ghosting)	T2W	Adjust phase–frequency direction (Figs. 4 and E5)	Minimises motion ghosting
	Hip prosthesis	DWI, DCE	Prefer 1.5 T (Fig. E6); adjust phase–frequency direction in focused/zoomed DWI (Fig. E7); include non–fat-sat GRE T1W and apply subtraction; add DCE if bpMRI is used (Fig. E8)	Reduces susceptibility distortion
	Protocol adherence	All	Experienced technologist supervision; confirm T2W, DWI/ADC, ± DCE coverage; balance the “MRI Quality Triangle” (SNR, resolution, scan time)	Ensures diagnostic adequacy
After scan (QA)	Artefact recognition	All	Immediate review of T2W, DWI, ADC; repeat sequences if inadequate	Prevents non-diagnostic scans; reduces recalls

Optimising prostate MRI quality: common artefacts, patient-related factors, and workflow strategies. This table summarises practical measures before, during, and after scanning to minimise artefacts and ensure diagnostic adequacy. Technical (protocol/hardware-related) issues and patient-related factors are combined with workflow checkpoints, highlighting the importance of patient preparation, technologist expertise, and immediate on-table quality assurance

*T2w* T2-weighted, *DWI* diffusion-weighted imaging, *ADC* apparent diffusion coefficient, *DCE* dynamic contrast-enhanced, *bpMRI* biparametric MRI, *SI* signal intensity, *SV* seminal vesicles, *PZ* peripheral zone, *SNR* signal-to-noise ratio *FOV* field of view, *GRE* Gradient Echo

## Step 2: See it right—interpretation, reporting and quality assurance

### Building expertise: structured training and certification

Interpretation quality is just as critical as acquisition quality, since variability in reader performance remains one of the key determinants of diagnostic accuracy [35–37]. The prostate MRI learning curve is steep, and diagnostic confidence strongly depends on the reader’s experience. According to consensus statements, including the ESUR/ESUI recommendations [15, 38–42]:

- A minimum of 50–150 supervised cases is required before performing independent reporting.
- Around 400 cases mark the threshold for a beginner.
- Typically, more than 1000 cases are needed to reach expert-level performance.
- Continued experience of more than 150 cases per year is recommended to maintain proficiency.

These thresholds serve as guidance for training programmes and certification pathways. Formalised training and quality assessment, such as the ESUR/ESR Certification in Prostate MRI, and continuing medical education are essential to maintain consistency across centres and sustain quality. To support broader dissemination and skill development, the ESUR Prostate MRI Working Group is planning ongoing educational initiatives, including hands-on image-quality workshops and focused sessions at major radiology meetings such as the ESUR Annual Symposium, along with dedicated prostate-focused training through the ESUR Prostate MRI Course. These activities aim to facilitate international knowledge transfer and encourage adoption of the three-step framework beyond the ESUR network. In the longer term, the framework may serve as a model that can be adapted across diverse healthcare systems, supporting broader international implementation of prostate MRI quality standards.

### Patient information and structured reporting

Accurate interpretation requires access to a core clinical dataset, ideally provided in the referral request or through the electronic health record. Essential details include PSA (and, where available, PSA kinetics), history of urinary tract infection, use of 5α-reductase inhibitors, prior androgen deprivation therapy, previous radiotherapy or focal ablation, intravesical Bacillus Calmette-Guérin, and prior biopsy results. Previous imaging is also critical, particularly for men in active surveillance or screening programmes [43].

Structured reporting templates ensure that these details, along with imaging findings, are consistently documented. PI-RADS v2.1 specifies that reports should include prostate volume (for PSA density), describe up to four suspicious lesions, and for each lesion provide location, size in millimetres, PI-RADS category, and features of extraprostatic extension. Lesion size should be measured according to PI-RADS rules: largest dimension on axial images (minimum), with sagittal/coronal measurements if larger; peripheral zone lesions on the ADC map; transition zone lesions on T2W; or, if needed, on whichever sequence best depicts the lesion. Optionally, three orthogonal dimensions or software-derived volume may be reported. The report should specify the sequence and series used, and a standardised diagram or sector map is recommended to facilitate communication with urologists.

Building expertise depends not only on case numbers and certification but also on continuous feedback linking imaging to pathology and outcomes—mechanisms best embedded within institutional quality assurance frameworks (see “Institutional quality assurance frameworks: from benchmarks to multidisciplinary integration” section).

### Institutional quality assurance frameworks: from benchmarks to multidisciplinary integration

#### PI-RADS 3 frequency as a practical indicator

The proportion of examinations categorised as PI-RADS 3 offers a pragmatic, biopsy-independent indicator of quality. The frequency of PI-RADS 3 reflects both the reader’s confidence and image quality. PCa detection rates, on the other hand, are strongly influenced by referral patterns and clinical decision-making.

Reported PI-RADS 3 rates vary considerably. A persistently high institutional PI-RADS 3 rate should first trigger a formal audit of technical image quality using an objective tool like PI-QUAL v2. This approach addresses the root cause (i.e., suboptimal image data) rather than just the symptom of interpretive uncertainty. Evidence confirms a direct correlation between better image quality (i.e., higher PI-QUAL scores) and improved diagnostic confidence, resulting in a significant reduction in indeterminate PI-RADS 3 calls. In the expert-driven, 3 T 4 M trial, PI-RADS 3 lesions appeared in 6% and 11% of cases, for mpMRI and bpMRI, respectively [44].

These considerations apply primarily to the setting for early detection, where the prevalence of csPCa ranges from 30 to 50% [45]. In population-based screening with lower disease prevalence, an optimal “recall rate” for PI-RADS 3 has not been established. Determining these thresholds requires prospective studies balancing diagnostic benefits with downstream tests, similar to other cancer screening programmes.

Practical strategies to ensure an appropriate PI-RADS 3 level include expert re-review, linking PI-RADS 3 cases with histopathological feedback, selective use of DCE-MRI by less experienced readers, and institutional audits that track PI-RADS 3 proportions over time [46–49].

Finally, it is essential to score PI-QUAL 1 examinations (i.e., insufficient quality) as non-diagnostic rather than assigning a PI-RADS 3 score.

#### Feedback, discrepancy reconciliation, and multidisciplinary integration

High-quality prostate MRI requires feedback loops that extend beyond image acquisition and reporting volumes. Reporting accuracy must be judged against patient outcomes, through structured discrepancy audits and multidisciplinary case reviews with urologists, pathologists and oncologists. Linking interpretations to biopsy or prostatectomy findings—particularly in centres with targeted biopsy or whole-mount correlation—ensures accountability and identifies areas for improvement.

When MRI results diverge from pathology—for example, a PI-RADS 5 lesion with negative biopsy (Fig. E9) or PI-RADS 1–2 with biopsy-proven csPCa—formal correlation is mandatory to determine whether the discrepancy reflects acquisition issues, interpretive error, or inherent MRI limitations. Embedding such reconciliation in QA dashboards, discrepancy meetings, or multidisciplinary discussions strengthens protocols, reduces unnecessary biopsies and overdiagnosis, and ultimately increases confidence in prostate MRI [50, 51]. Beyond technical accuracy, QA should also assess downstream clinical impact—for example, whether reports lead to appropriate biopsy recommendations. Incorporating these elements ensures that MRI reporting is not only internally consistent but also fully integrated into the wider prostate cancer care pathway [1, 43].

#### Collaborative quality improvement initiatives

Recently, the ACR launched the Prostate MR Image Quality Improvement Collaborative, uniting multidisciplinary teams to standardise prostate MRI protocols (using the PI-QUAL score) and implement targeted interventions like protocol refinement, patient preparation, and education [52]. This increased with the proportion of good-quality examinations from 67% to 87%, showing system-level QA initiatives improve image quality and protocol adherence [53].

#### Key institutional QA metrics

At the institutional level, QA efforts should focus on a few measurable benchmarks (Table 3): PI-QUAL scores, PI-RADS score 3 call rates, detection rates of csPCa (Grade Group ≥ 2), and radiology-pathology discrepancy audits. These metrics should inform feedback systems, like double-reading discordant cases, discrepancy meetings, and dashboards. QA must remain flexible, integrating acquisition, interpretation, and outcomes into a continuous quality-improvement cycle to enhance diagnostic consistency and institutional accountability. In some settings, operator experience—often reflected by the proportion of positive MRI-targeted biopsies using fusion or in-bore approaches—may serve as an additional quality indicator, linking interpretive accuracy with procedural performance.

**Table 3:** Proposed quality indicators and corrective actions for prostate MRI

Indicator	Rationale	Suggested benchmark	Corrective action if out of range
PI-RADS 3 frequency	Surrogate of interpretive competence	~10–20%	• If **below 10%**: risk of under-calling equivocal findings → review reader thresholds and objectivity. • If **above 20%**: risk of overuse in non-diagnostic studies → review image quality, retrain readers, and designate such scans as “non-diagnostic” (PI-QUAL score 1) rather than defaulting to PI-RADS 3. • Overuse of PI-RADS 3 can also reflect less experienced readers using it as a “safety net”, underscoring the need for expert second reading and structured internal review programmes.
csPCa detection rate (PPV)	Clinical impact measure	At least ~50% when adjusted for local prevalence; should align with trial benchmarks. PPV should be reported both at the patient level and per target biopsy.	Audit diagnostic pathway, ensure biopsy correlation, and integrate MDT radiology/pathology reconciliation data.
PI-QUAL v2 distribution	Acquisition quality	≥ 80% of scans at level 3	Review scanner calibration and protocols; implement targeted interventions (e.g., as demonstrated in the GLIMPSE study).
Radiology–pathology discrepancy audit rate	Feedback mechanism	Continuous monitoring	Conduct MDT-based reviews of discordant cases; refine reader training and acquisition protocols.

This table summarises potential quality indicators that can be monitored at the institutional level, their rationale, suggested benchmarks, and corrective actions if values fall outside expected ranges. Indicators span interpretive confidence (PI-RADS 3 rates), clinical impact (csPCa detection rate), acquisition quality (PI-QUAL), and continuous feedback mechanisms (radiology/pathology discrepancy audits). These benchmarks are not prescriptive but provide a framework to guide local QA programmes and support iterative improvement, with expected values varying according to local disease prevalence, referral pathways (screening vs diagnostic referrals), biopsy strategy (targeted-only vs targeted plus systematic biopsy), and institutional case-mix (type of patients typically scanned at a given centre)

*PI-RADS* Prostate Imaging-Reporting and Data System, *PI-QUAL* Prostate Imaging Quality score, *csPCa* clinically significant prostate cancer, *PPV* positive predictive value, *QA* quality assurance, *MDT* multidisciplinary team

### The QA workforce: beyond the radiologist

Technologists and radiographers serve as the frontline in QA, handling patient preparation and spotting artefacts in real time. On-table recognition of suboptimal sequences enables on-table correction, such as switching the phase-encoded direction, with on-table QA [54]. Experienced technologists can evaluate prostate MRI quality with agreement levels similar to radiologists, demonstrating their ability to detect problems and make immediate corrections [55]. This offers a solid, evidence-based reason for empowering technologists to perform on-table QA and repeat inadequate sequences before the patient leaves the scanner.

Empowering technologists to flag deviations and repeat inadequate sequences is essential to prevent non-diagnostic examinations. Future certification programmes for technologists and radiographers in prostate MRI could standardise training and support a capable workforce, especially if MRI is used in population-based screening.

MRI physicists safeguard technical robustness by calibrating scanners, troubleshooting hardware/software, and maintaining cross-platform consistency.

### Radiologist-patient communication

Radiologist–patient communication is an often-overlooked but crucial aspect of prostate MRI quality. The ACR Prostate Collaborative identified inadequate communication as a leading cause of poor compliance with preparation instructions. Providing clear, timely guidance significantly improves image quality. Structured reports, patient-friendly summaries, and selective consultations enhance transparency, accountability, and trust in imaging-driven care. When patients understand why surveillance may be preferred over biopsy, they are more likely to accept MRI-based monitoring.

Perlis et al introduced the Patient-Centred Prostate MRI Report, which improved comprehension and engagement compared with conventional reports [56]. A pilot trial confirmed that simplified, patient-oriented summaries reduced decisional conflict and clarified management pathways [57]. Studies by Merriel et al and Sutherland et al found that men, particularly those on active surveillance, valued clear explanations of MRI findings but often encountered fragmented communication [58, 59]. Brief pre-procedural interactions between radiologists and patients were associated with reduced anxiety and greater satisfaction [60]. Limitations include time constraints in busy practices and variation in institutional expectations for radiologists.

## Step 3: Improve and innovate—the future of prostate MRI quality assurance

**Fig. 5:**
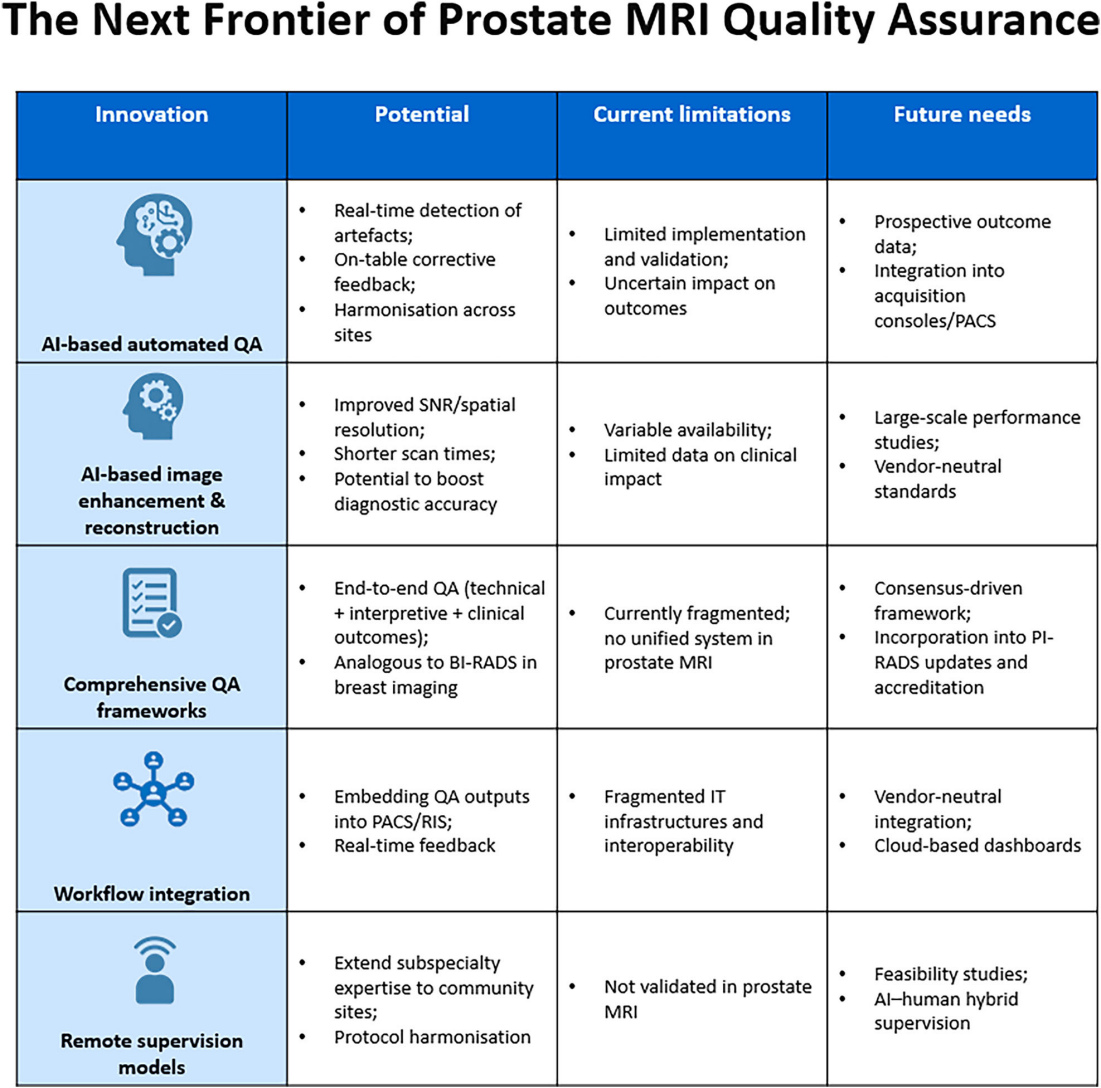
Innovations shaping the future of prostate MRI quality assurance. These include AI-driven tools, image enhancement and reconstruction, comprehensive QA frameworks, workflow integration, and remote supervision models. Each is outlined by its potential, current limitations, and future needs. AI, artificial intelligence; QA, quality assurance; PACS, picture archiving and communication system; RIS, radiology information system; SNR, signal-to-noise ratio; BI-RADS, Breast Imaging Reporting and Data System; PI-RADS, Prostate Imaging-Reporting and Data System

**Fig. 6:**
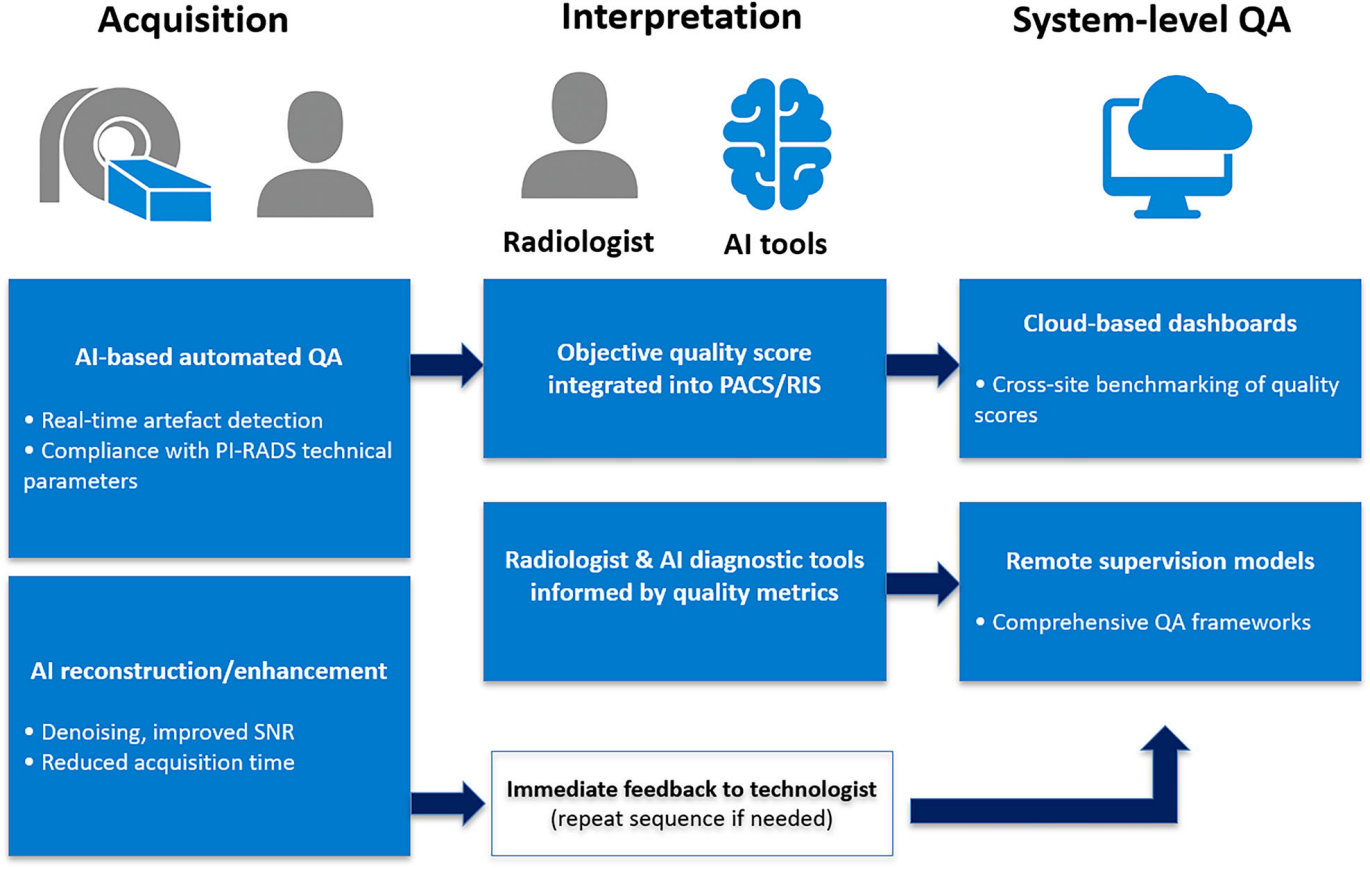
The future ecosystem of prostate MRI quality assurance. A stepwise model is illustrated, spanning three domains: acquisition, where AI-based automated quality assurance (QA) enables real-time artefact detection and compliance with PI-RADS technical standards, alongside AI-driven image reconstruction for denoising, improved signal-to-noise ratio (SNR), and reduced acquisition time; interpretation, where radiologists and AI tools are supported by objective quality scores integrated into PACS/RIS, with feedback loops to technologists for immediate sequence correction; and system-level QA, where cloud-based dashboards allow cross-site benchmarking of quality scores and remote supervision models extend expert oversight, harmonise protocols, and support comprehensive institutional QA frameworks. Together, these innovations illustrate how automation, AI, and organisational models may converge to sustain reproducible and high-quality prostate MRI. PI-RADS, Prostate Imaging-Reporting and Data System; QA, quality assurance; SNR, signal-to-noise ratio; PACS, picture archiving and communication system; RIS, radiology information system

Prostate MRI technical standards and structured quality scoring are now in place through PI-RADS and PI-QUAL. The next step is to move towards real-time, objective, and system-wide QA. Advances in artificial intelligence (AI), deep learning–based reconstruction, and organisational innovation are likely to shape this next frontier (Figs. 5 and 6).

### Automated quality assurance with AI

Manual QA is time-consuming and inconsistently applied. Automated tools can verify acquisition parameters, detect artefacts, and generate objective quality scores with accuracy comparable to inter-reader agreement [61–64]. Crucially, these systems promise on-table feedback, enabling technologists to repeat insufficient sequences before the patient leaves the scanner. This reduces recalls, improves diagnostic performance and consistency across institutions, and saves time.

In addition to fully automated systems, semi-automated AI-assisted platforms for PI-QUAL–based image quality assessment are emerging, offering more consistent application of quality criteria and the potential to support on-table decision-making. Early commercial deployments and prospective evaluations of semi-automated PI-QUAL software demonstrate feasibility for real-world integration and may serve as a practical bridge between manual scoring and fully autonomous AI-driven quality assurance [65, 66]. As these systems evolve, they are expected to integrate alongside computer-aided diagnostic tools, supporting both acquisition and interpretation and informing future training pathways for readers adopting AI-enabled workflows.

### Image quality as a prerequisite for diagnostic AI

AI also supports image acquisition directly through deep learning reconstruction techniques that denoise, boost SNR, improve spatial resolution, shorten scan times, and enhance overall quality [67, 68]. These tools are increasingly available from vendors and developers.

At the same time, diagnostic AI models for cancer detection depend heavily on high-quality images—often more so than human readers, whose experience and judgement may compensate for imperfections. Their performance declines in the presence of real-world artefacts or when technical parameters diverge from training datasets. As clinical reliance on AI grows, strict adherence to acquisition standards will be essential to ensure reliable outputs.

Although vendors are beginning to integrate quality scoring into commercial AI platforms, their effect on acquisition quality and reporting consistency has yet to be clinically validated. While the impact of image quality on human readers is well established [18], its influence on AI diagnostics remains insufficiently studied and requires further research to guarantee trustworthy results.

### Towards comprehensive QA frameworks

Technical adequacy is only one part of a high-quality imaging pathway. Other subspecialties, such as breast radiology, demonstrate the importance of comprehensive frameworks. The BI-RADS atlas [69] combines technical standards with accreditation, interpretive auditing, and structured follow-up requirements.

A similar approach in prostate MRI could unify currently disparate efforts. A comprehensive, BI-RADS–like system would logically integrate PI-RADS, PI-QUAL [25], and other specialised reporting systems such as the Prostate Cancer Radiological Estimation of Change in Sequential Evaluation (PRECISE) and Prostate Imaging for Recurrence Reporting (PI-RR) scores [9, 70–72] within a single conceptual framework.

### Workflow integration

For QA and AI-based reconstruction to succeed, integration into the daily workflow is critical. Embedding QA outputs into Picture Archiving and Communication System (PACS) enables radiologists to incorporate results directly into reports. Displaying them on acquisition consoles or Radiology Information Systems allows technologists to correct issues in real time. At a broader level, cloud-based dashboards could support cross-site benchmarking, harmonise quality across networks, and facilitate multicentre studies [63].

### Emerging organisational innovations

Technological progress must be matched by organisational innovation. Remote supervision models, or “virtual command centres”, are being piloted to extend subspecialty expertise across sites. These frameworks enable experts to guide technologists in real time, harmonise protocols, reduce variability, and expand access to high-quality prostate MRI in resource-limited settings [73, 74]. While not yet validated specifically for prostate MRI, they illustrate how structural changes can complement AI-driven tools. For example, regulatory changes by the US Centres for Medicare & Medicaid Services recently extended remote supervision for contrast administration through 2025, underscoring the potential of such innovations. Importantly, regulatory frameworks governing remote supervision vary by jurisdiction, and this US-based example is intended to illustrate the broader concept rather than a universally applicable policy. These models have particular relevance for smaller and non-academic sites, where remote supervision, AI-supported feedback, shared QA dashboards, and off-site expertise can compensate for limited on-site subspecialty presence and facilitate broader implementation of prostate MRI quality standards.

## Conclusion

Prostate MRI has transformed prostate cancer care, but its impact depends on consistent quality across the pathway. The Quality Improvement Subgroup of the ESUR Prostate MRI Working Group proposes a three-step approach (‘Build it right’; ‘See it right’; ‘Improve and innovate’), emphasising acquisition, interpretation, and future integration of AI-driven tools (Fig. 1, Table 4). We believe that embedding these principles into routine practice will ensure prostate MRI remains a robust, reproducible, and high-quality diagnostic tool that continues to evolve with patient-centred care.

**Table 4:** ESUR Prostate MRI Working Group perspectives on improving MRI quality

Step	ESUR perspectives and practical suggestions
Step 1: Build it right—acquisition quality	1. Adhere to PI-RADS v2.1 minimum technical standards—essentials: - T2W (3-mm slices), - DWI (≤ 4-mm slices, high b ≥ 1400, ADC from ≥ 2 b-values up to 1000), - DCE (3-mm slices, temporal resolution ≤ 15 s, T1-weighted fat-suppressed or subtraction). 2. Ensure coverage includes prostate, seminal vesicles (straight axial or oblique axial plane perpendicular to the prostate long axis); Large-FOV T1W (pre-/post-contrast per practice) or 3D isotropic T2W extending to the aortic bifurcation for nodal assessment (optional). 3. Apply PI-QUAL systematically before PI-RADS scoring. 4. Follow standardised patient preparation (bowel emptying, micro-enema, fasting, avoid caffeine/nicotine, spasmolytics if not contraindicated, anxiety reduction). 5. Mitigate common artefacts proactively (adjust FOV, bandwidth, matrix; adapt phase–frequency direction; use non–fat-sat GRE in hip prosthesis; sedation if severe claustrophobia). 6. Perform on-table QA and repeat inadequate sequences immediately.
Step 2: See it right—interpretation, reporting, and QA	7. Achieve competence through ≥ 50–150 supervised cases before independent reporting; keep > 150 cases/year for maintaining proficiency. 8. Engage in structured training (e.g., ESUR/ESR certification) and CME. 9. Monitor PI-RADS/Likert 3 frequency (target 10–20%); classify poor-quality scans as non-diagnostic (PI-QUAL score 1) rather than PI-RADS 3. 10. Include the core clinical information dataset and use structured reporting templates. 11. Establish institutional QA dashboards tracking PI-QUAL distribution, PI-RADS/Likert 3 frequency, csPCa detection, and radiology-pathology discrepancies. 12. Obtain histopathology feedback via multidisciplinary review. 13. Conduct double-reading or discrepancy meetings for outliers. 14. Perform regular scanner calibration; adopt phantom-based QA. 15. Consider participating in institutional/multicentre accreditation (e.g., ACR DICOE, ESUR initiatives). 16. Radiologist-patient communication (patient-friendly summaries, brief consultations) is promising but limited by time, workflow, and reimbursement barriers.
Step 3: Improve and innovate—future directions	17. Explore AI-based QA for real-time compliance and artefact detection. 18. Evaluate AI reconstruction to enhance SNR and reduce noise. 19. Support development of vendor-neutral, PACS-integrated QA dashboards. 20. Investigate how image quality impacts AI performance in prostate cancer detection. 21. Pilot cloud-based benchmarking and remote supervision models to harmonise protocols and expand access to expertise.

This table outlines practical suggestions of the ESUR Prostate MRI Working Group on strategies to improve MRI quality across three domains: (1) building robust acquisition, (2) ensuring reliable interpretation, reporting, and institutional QA, and (3) innovating through AI, automation, and new organisational models. The numbered items represent sequential, consensus-based practical suggestions that together form a roadmap for sustaining excellence in prostate MRI

*PI-RADS* Prostate Imaging-Reporting and Data System, *PI-QUAL* Prostate Imaging Quality score, *T2W* T2-weighted, *DWI* diffusion-weighted imaging, *ADC* apparent diffusion coefficient, *DCE* dynamic contrast-enhanced imaging, *mpMRI* multiparametric MRI, *FOV* field of view, *QA* quality assurance, *CME* continuing medical education, *csPCa* clinically significant prostate cancer, *ACR DICOE* American College of Radiology Diagnostic Imaging Center of Excellence, *PACS* picture archiving and communication system, *SNR* signal-to-noise ratio, *ESR* European Society of Radiology, *ESUR* European Society of Urogenital Radiology

## Supplementary information

Supplementary information

## Abbreviations

ACR: American College of Radiology
ADC: Apparent diffusion coefficient
bpMRI: Biparametric
cs: Clinically significant
DWI: Diffusion-weighted imaging
ESUR: European Society of Urogenital Radiology
mpMRI: Multiparametric MRI
PACS: Picture Archiving and Communication System
PCa: Prostate cancer
PI-QUAL: Prostate Imaging Quality
PI-RADS: Prostate Imaging Reporting and Data System
SNR: Signal-to-noise ratio
