# Supplementary material for: Prostate MRI quality improvement: a Roadmap from the ESUR Prostate MRI Working Group
Source: Eur Radiol. 2026 Mar 13;36(7):5728–44. doi: 10.1007/s00330-026-12395-w (PMC13282344; doi:10.1007/s00330-026-12395-w)
Supplement: Supplementary file 1 — Supplementary information [file 330_2026_12395_MOESM1_ESM.pdf]

Prostate MRI Quality Improvement: A Roadmap from the ESUR Prostate MRI Working Group

ELECTRONIC SUPPLEMENTARY MATERIAL

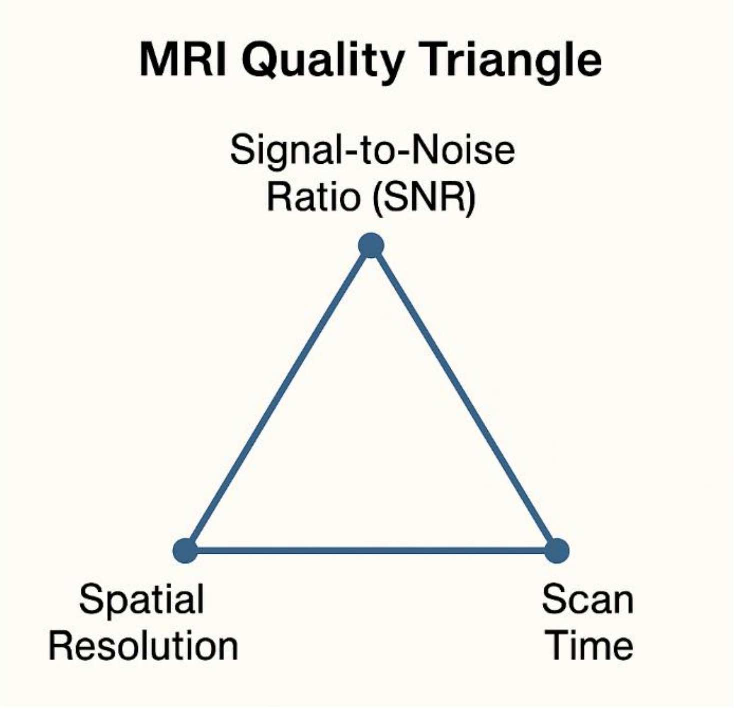

Figure E1. MRI Quality Triangle. The three fundamental determinants of image quality in prostate MRI—signal-to-noise ratio (SNR), spatial resolution, and scan time—are represented as a triangle. Optimising one parameter inevitably affects the others: increasing spatial resolution reduces SNR or requires longer acquisition times; improving SNR may necessitate longer scans or reduced resolution; and shortening scan time typically comes at the cost of lower SNR or resolution. Understanding and balancing this "MRI Quality Triangle" is critical for tailoring protocols to clinical priorities, whether maximising lesion conspicuity, minimising artefacts, or ensuring patient comfort.

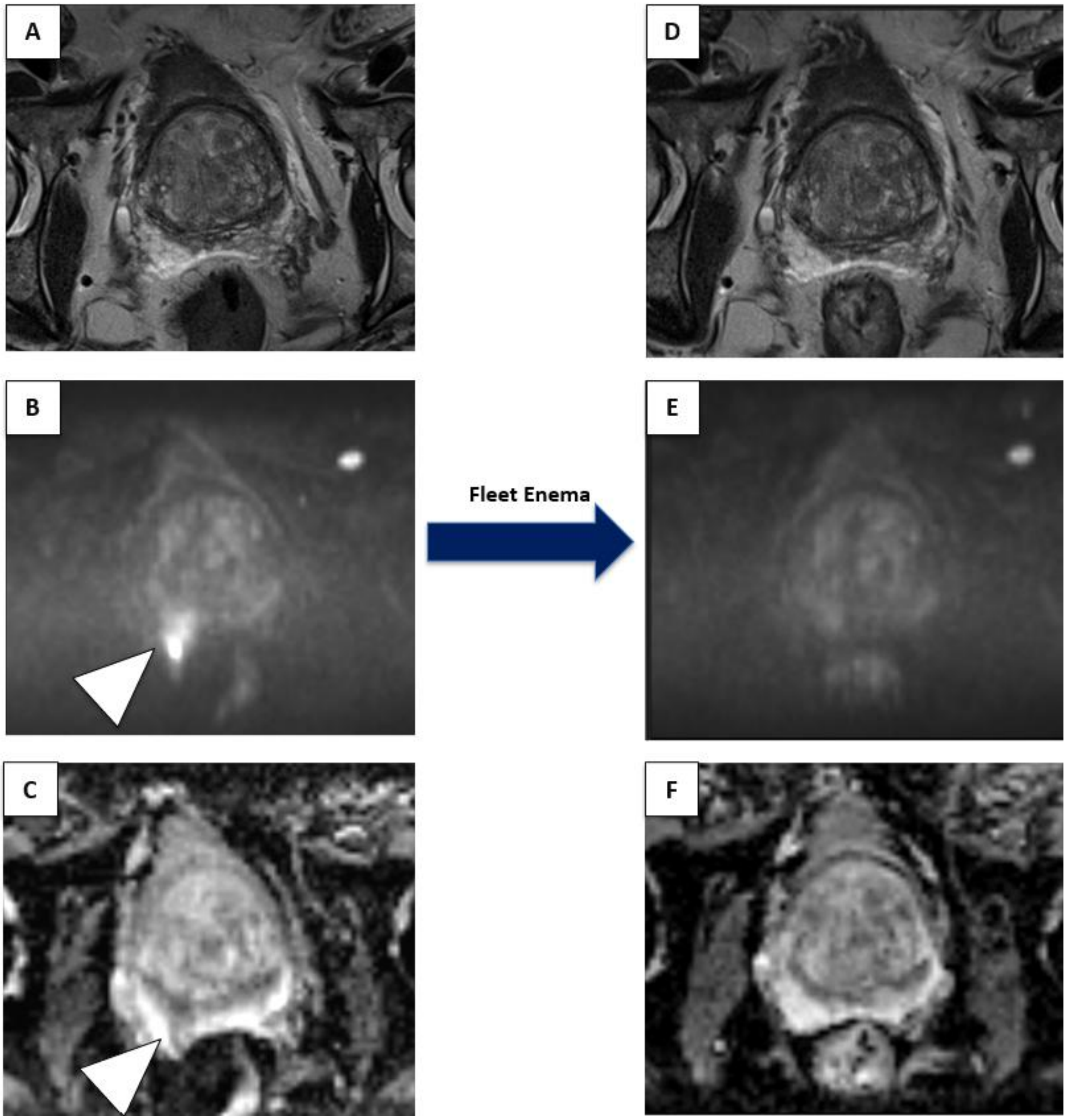

Figure E2. Effect of bowel preparation on false-positive findings. (A–C) Initial MRI in a patient with rising PSA (13.2 ng/mL, PSA density 0.15) shows rectal gas artefact. Axial T2-weighted image (A) is unremarkable, but high b-value DWI (B, arrowhead) and ADC map (C, arrowhead) demonstrate an apparent focus of diffusion restriction in the right base, mimicking tumour. No corresponding abnormality was seen on DCE (not shown). (D–F) Repeat MRI after fleet enema confirms resolution of the artefact, with no true lesion identified. This case illustrates how bowel preparation can reduce artefacts and help avoid false-positive interpretations on prostate MRI.

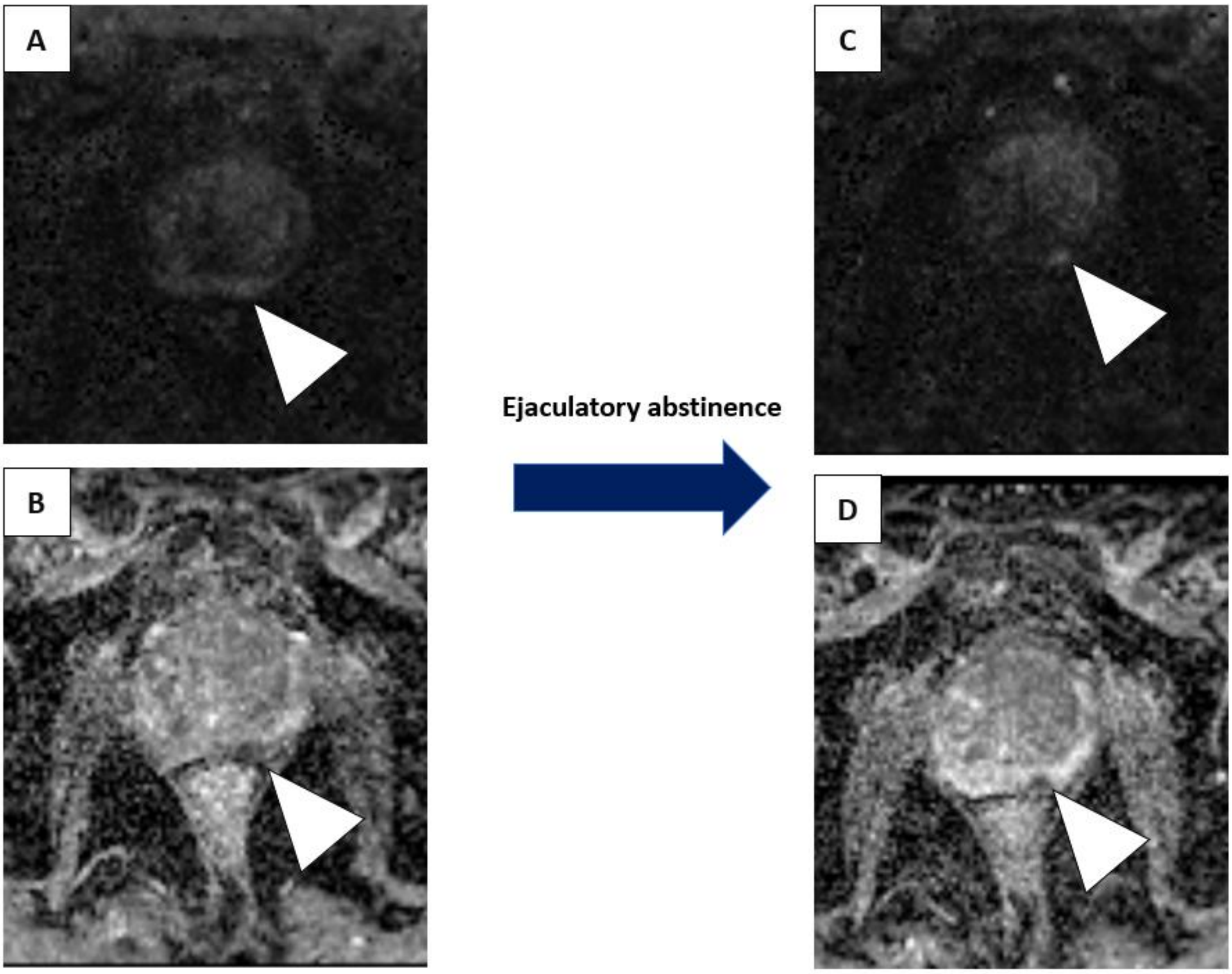

Figure E3. **Effect of ejaculatory abstinence on prostate MRI.** (A, B) Diffusion-weighted imaging (DWI) and apparent diffusion coefficient (ADC) map acquired without abstinence show an “empty” peripheral zone, limiting lesion conspicuity. (C, D) Repeat imaging after three days of abstinence demonstrates a more homogeneous, filled peripheral zone, with clear identification of a PI-RADS 4 lesion in the left dorsal region (arrowheads). This case illustrates how ejaculatory status can alter peripheral zone appearance and highlights the importance of patient preparation in prostate MRI interpretation**.**

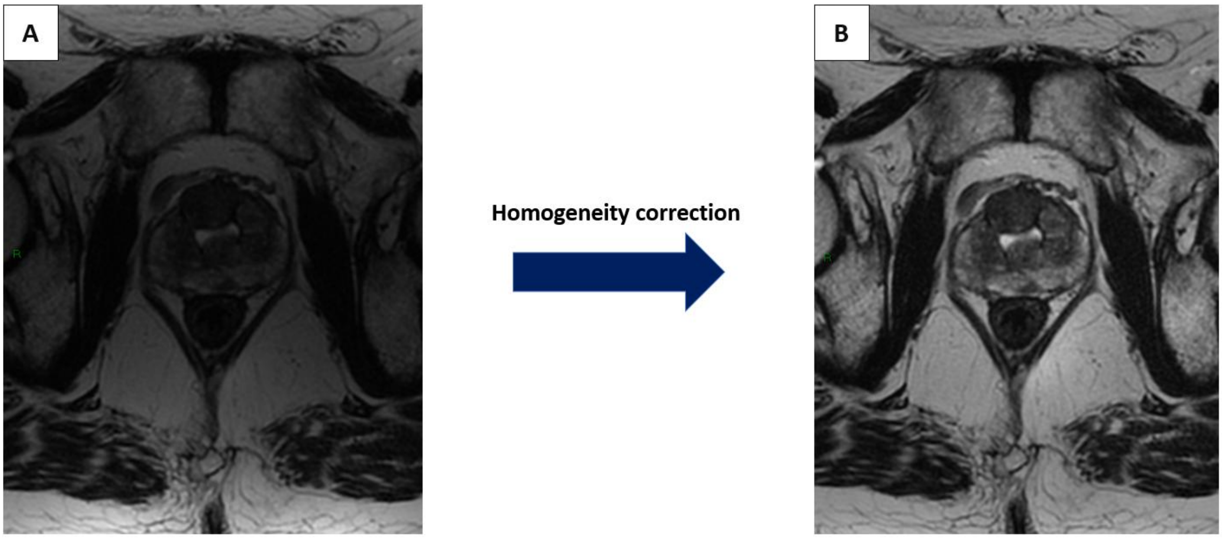

Figure E4. Effect of post-processing for homogeneity correction. (A) Axial T2-weighted image at 3T demonstrates signal inhomogeneity with low signal intensity across the prostate region, limiting uniform assessment. (B) After post-processing homogeneity correction, signal distribution is more uniform, resulting in improved visualisation of the gland. This example illustrates how homogeneity correction can enhance image quality and increase diagnostic confidence in prostate MRI.

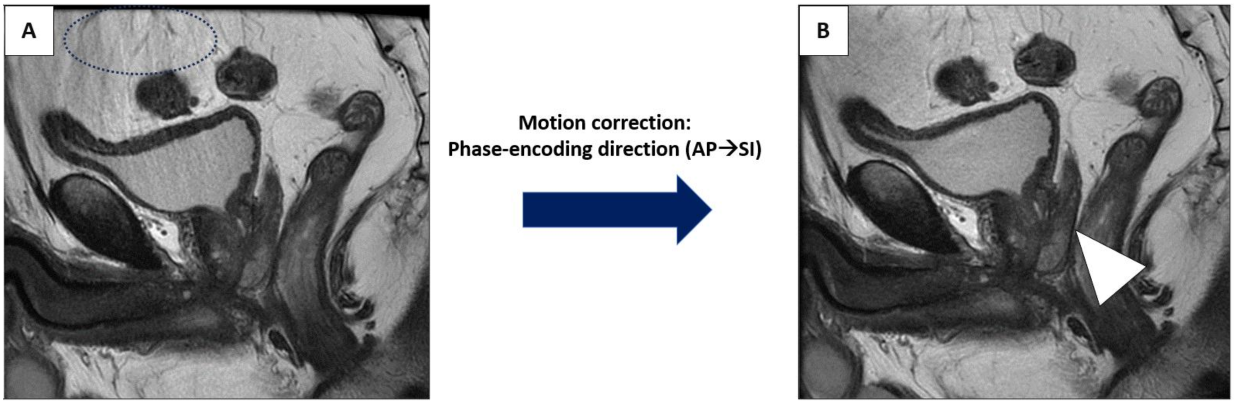

Figure E5. Effect of phase-encoding direction on motion artifacts in prostate MRI. (A) Sagittal T2-weighted image acquired with anterior–posterior (AP) phase encoding shows respiratory ghosting (dashed circle). (B) Re-acquisition with superior–inferior (SI) phase encoding removes these artefacts, resulting in sharper delineation of the posterior prostatic capsule (arrowhead) and clearer visualisation of adjacent structures. This example highlights how adjusting phase-encoding direction can reduce motion artefacts and improve diagnostic image quality.

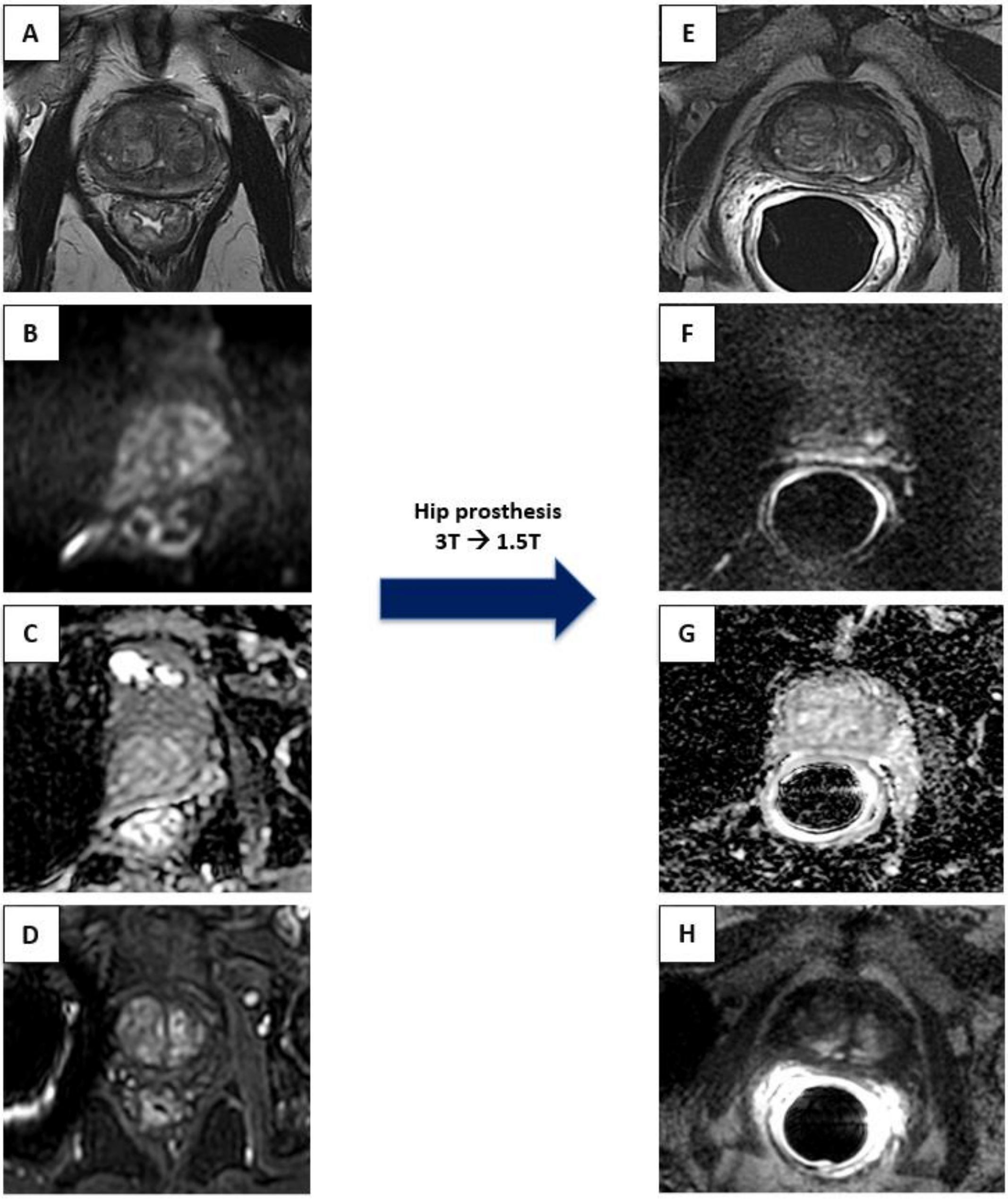

Figure E6. Effect of field strength in a patient with a right hip prosthesis. (A–D) 3T MRI demonstrates severe susceptibility artefacts, particularly on diffusion-weighted imaging (B) and the ADC map (C), precluding reliable assessment of the prostate. (E–H) Repeat imaging at 1.5T markedly reduces artefacts and restores diagnostic quality, with an endorectal coil also used in this examination. Notably, the dynamic contrast-enhanced (DCE) sequence (D, H) remains largely unaffected at both field strengths. This case illustrates the importance of adapting field strength in patients with metallic implants to optimise prostate MRI.

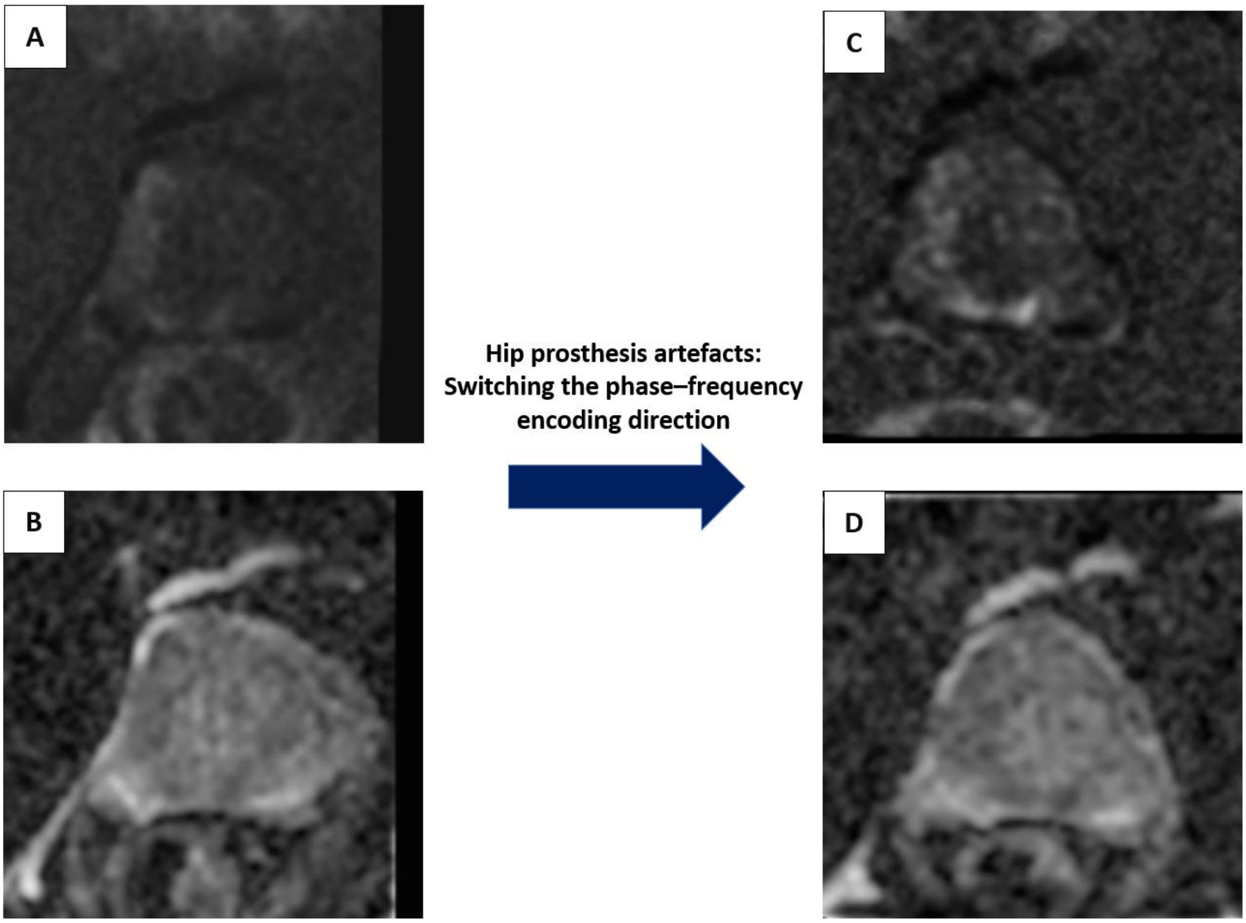

Figure E7. Effect of changing the phase–frequency encoding direction on diffusion-weighted imaging (DWI) in a patient with a right-sided hip prosthesis. (A, B) DWI and ADC map acquired with the standard phase–frequency direction demonstrate severe distortion and signal loss adjacent to the prosthesis, obscuring the prostate. (C, D) Repeat acquisition with anterior–posterior (AP) phase encoding (after switching phase and frequency directions) markedly reduces susceptibility artefact and restores prostate visualisation. This example highlights how modifying the phase–frequency direction can mitigate metal-related artefacts and improve diagnostic quality in prostate MRI.

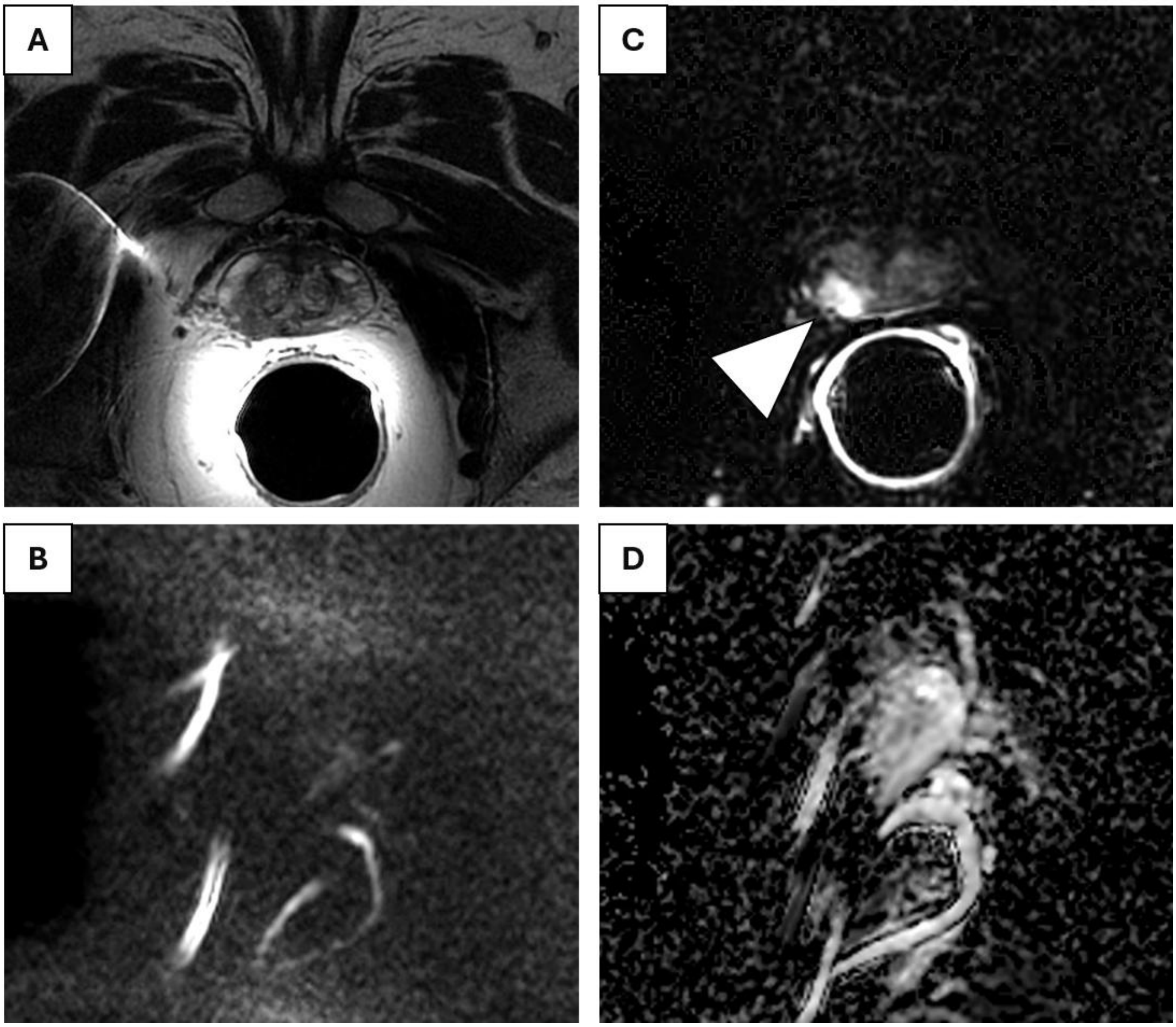

Figure E8. Value of dynamic contrast–enhanced (DCE) imaging as a "safety-net" sequence. In a patient with a right hip prosthesis, severe susceptibility artefacts on 1.5T diffusion-weighted imaging (B) and the apparent diffusion coefficient (ADC) map (D) preclude reliable lesion assessment, despite the use of an endorectal coil. However, DCE imaging (C) demonstrates a focal area of hyperenhancement in the right prostate (C, arrowhead), corresponding to ISUP grade group 4 (Gleason 4+4) prostate cancer. This case highlights the critical role of DCE as a complementary sequence when DWI is nondiagnostic.

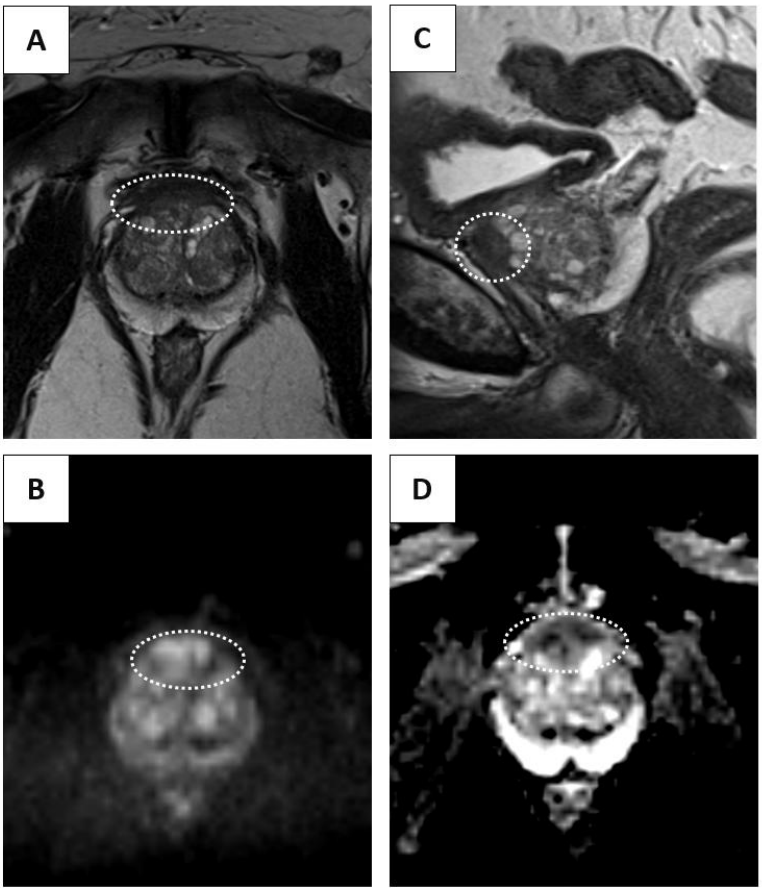

Figure E9. Importance of multidisciplinary review in the setting of discordant imaging–pathology findings. A 71-year-old male with a PI-RADS 5 anterior midgland/base lesion. (A) Axial T2-weighted, (B) (B) high b-value diffusion-weighted, (C) sagittal T2-weighted, and (D) apparent diffusion coefficient (ADC) map images are shown, with the lesion circled. Despite a prior negative targeted biopsy, the patient's persistently rising PSA prompted multidisciplinary discussion, which raised concern for sampling error. Repeat MRI-targeted biopsy confirmed ISUP grade group 2 prostate cancer (20% pattern 4), and the patient subsequently underwent radical prostatectomy. This case underscores the value of multidisciplinary review and repeat biopsy consideration when MRI findings remain highly suspicious despite negative histology.
